# Association between estimated glomerular filtration rate and sodium excretion in urine of African descendants in Brazil: a population-based study

**DOI:** 10.1590/2175-8239-JBN-3864

**Published:** 2018-05-07

**Authors:** Elisângela Milhomem dos Santos, Dyego José de Araújo Brito, Ana Karina da Cunha Teixeira França, Joyce Santos Lages, Alcione Miranda dos Santos, Natalino Salgado

**Affiliations:** 1 Universidade Federal do Maranhão, Departamento de Enfermagem, São Luís, MA, Brasil.; 2 Universidade Federal do Maranhão, Departamento de Nefrologia do Hospital Universitário, São Luís, MA, Brasil.; 3 Universidade Federal do Maranhão, Departamento de Ciências Fisiológicas, São Luís, MA, Brasil.; 4 Universidade Federal do Maranhão, Departamento de Saúde Pública, São Luís, MA, Brasil.

**Keywords:** Sodium, Glomerular Filtration Rate, Chronic Kidney Disease, Vulnerable Population, Sódio, Taxa de Filtração Glomerular, Doença Renal Crônica, Populações Vulneráveis

## Abstract

**Introduction:**

Excessive salt intake is a risk factor for the development of chronic kidney disease (CKD). Objective: To evaluate the association between estimated glomerular filtration rate (eGFR) and sodium excretion in urine samples of Brazilians of African ancestry.

**Methods:**

Cross-sectional, population-based study of 1,211 Brazilians of African ancestry living in Alcântara City, Maranhão, Brazil. Demographic, nutritional, clinical, and laboratory data were analyzed. The urinary excretion of sodium was estimated using the Kawasaki equation. Calculations of eGFR were based on the Chronic Kidney Disease Epidemiology Collaboration equation. Multivariate linear-regression model was used to identify the relationship between sodium excretion and eGFR.

**Results:**

Mean age was 37.5±11.7 years and 52.8% were women. Mean urinary excretion of sodium was 204.6±15.3 mmol/day and eGFR was 111.8±15.3 mL/min/1.73m^2^. According to multivariate linear regression, GFR was independently correlated with sodium excretion (β=0.11; p<0.001), age (β=-0.67; p<0.001), female sex (β=-0.20; p<0.001), and body mass index (BMI; β=-0.09; p<0.001).

**Conclusions:**

The present study showed that age, female sex, BMI, and correlated negatively with eGFR. Sodium excretion was the only variable that showed a positive correlation with eGFR, indicating that high levels of urinary sodium excretion may contribute to hyperfiltration with potentially harmful consequences.

## RESUMO

**Introdução:**

O consumo excessivo de sal é um fator de risco para o desenvolvimento de doença renal crônica (DRC). Objetivo: Avaliar a associação entre taxa de filtração glomerular estimada (eGFR) e excreção urinária de sódio em amostra isolada de urina de brasileiros de ascendência africana.

**Métodos:**

Trata-se de um estudo transversal de base populacional que incluiu 1.211 brasileiros de ascendência africana que vivem na cidade de Alcântara, no Maranhão. Foram analisados dados demográficos, nutricionais, clínicos e laboratoriais. A excreção urinária de sódio foi estimada usando a equação de Kawasaki. Os cálculos da TFGe foram realizados por meio da equação do Chronic Kidney Disease Epidemiology Collaboration. O modelo de regressão linear multivariada foi utilizado para identificar a relação entre excreção de sódio e TFGe.

**Resultados:**

A idade média foi de 37,5 ± 11,7 anos e 52,8% dos participantes eram mulheres. A média da excreção urinária de sódio, ao invés de excreção urinária média foi de 204,6 ± 15,3 mmol/dia e a TFGe foi de 111,8 ± 15,3 mL/min/1,73 m^2^. A regressão linear multivariada mostrou que a TFG correlacionou-se independentemente com a excreção de sódio (β = 0,11; *p* < 0,001), idade (β = -0,67; *p* < 0,001), sexo feminino (β = -0,20; *p* < 0,001) e índice de massa corporal (IMC; β = -0,09; *p* < 0,001).

**Conclusões:**

O presente estudo mostrou que idade, sexo feminino e IMC correlcionaram-se negativamente com TFGe. Ao negativamente correlacionados com TFGe. Excreção de sódio foi a única variável que mostrou correlação positiva com TFGe, indicando que a alta excreção urinária de sódio pode determinar um quadro de hiperfiltração, acarretando consequências adversas para a função renal a longo prazo.

## INTRODUCTION

High sodium intake is one of the main risk factors for the development of diseases, such as stroke, left ventricular hypertrophy, chronic kidney disease (CKD), and hypertension.^1^ In turn, hypertension is one of the major causes of CKD. High salt intake may have deleterious effects on glomerular hemodynamics, inducing hyperfiltration and increasing the filtration fraction and glomerular pressure. This may be particularly important in certain groups such as elderly, obese, diabetic or black patients, who have a high prevalence of salt-sensitivity.^2^ Consistent decreases in proteinuria have also been observed with sodium restriction, regardless of changes in BP.^3^

Sodium excretion in a 24-h urinary sample is considered the gold standard for the evaluation of sodium intake. However, the difficulties associated with the accuracy of such urine collection may interfere with the results, especially in population studies. Thus, the estimate of sodium intake from the collection of random urine, at a population level, has been increasingly used as a convenient and affordable alternative. Several formulas have been proposed to convert sodium in a random single-sample urine into a 24-h excretion estimation, using creatinine values to adjust for urinary concentration.^4^

Kawasaki et al.^5^ and Tanaka et al.^6^ proposed formulas based on random urine samples in a Japanese population. Estimates of urinary sodium excretion using these formulas were close to actual 24-h excretion values. The authors concluded that the collection of random urine is a suitable alternative to 24-h urine collection in population surveys.

Studies in Latin America to assess the effects of increased sodium excretion on renal function in ethnic minority groups, including Afro-descendants, are scarce. Thus, the aim of the present study was to evaluate the association between estimated glomerular filtration rate (eGFR) and sodium excretion in urine samples of Brazilians of African ancestry who live in a Northeast city in Brazil.

## MATERIALS AND METHODS

A cross-sectional population-based study was conducted on Brazilians of African ancestry living in the city of Alcântara, state of Maranhão, northeastern Brazil. The data in this study are part of the Prevalence of Chronic Kidney Disease in Quilombo Communities of Alcântara, Maranhão State (PREVRENAL) survey. The survey aimed to determine the prevalence of chronic kidney disease in that population from July 2012 to April 2013.

Participants were derived from communities known in Brazilian Portuguese as quilombos, which are communities of self-defined ethnic and racial group with their own historical trajectory, specific territorial relations, and presumption of descent from Afro-Brazilian slaves. In general, quilombos have a certain degree of geographical isolation and cultural practices that have strong roots in the past.^7^

The sample was obtained by probability sampling procedures, in two-stage cluster sampling of census sectors and households. In the first stage, 32 census sectors (quilombos) were selected randomly from 139 known existing quilombos. The numbers of households were obtained for the selected sectors. In the second stage, in order to reach the necessary sample size, households were randomly selected. From the selected households, all individuals aged 18 years or more were invited to participate in the study. The purpose of the study was explained to those who accepted, and they signed an Informed Consent (IC).

Exclusion criteria were individuals less than 18 years of age, pregnant women, those using immunosuppressive drugs or with thyroid disorders, and patients with the following (based on clinical history and physical examination): consumptive chronic diseases (cancer or acquired immunodeficiency syndrome), hematological diseases, autoimmune diseases, systemic or genitourinary tract infection, and chronic or acute kidney disease undergoing dialysis.

Sample size was calculated based on the power to detect correlations between GFR and sodium excretion, assuming a correlation between the two measures of at least 0.10, alpha error of 0.05 with a two-tailed test, and power of 0.90 to detect a large correlation. The calculated minimum sample size was 1,047 individuals. An additional 15% was added to compensate for eventual losses (n = 1,205).

The initial stage of the study comprised the collection of household data, through interviews using a structured questionnaire, including demographic and socioeconomic, lifestyle, previous medical history, medication consumption, use of health services.

For the evaluation of economic status, two factors were considered: the family's monthly income, categorized into multiples of minimum wage (which in Brazil at the time was approximately US $296), and the Criterion of Brazilian Economic Classification (CCEB), which divides the population into seven classes: A1 and A2, B1 and B2, C, D, and E, from best to worst economic status8. The education of individuals was categorized as ≤ 8 years and > 8 years of formal study.

After answering the questionnaire, the BP was measured on the right arm while the individual was seated, after resting for at least 5 min, using a digital sphygmomanometer (Omron(r) 705-IT, Japan), with an appropriate-sized cuff covering approximately 80% of the arm area. Three measurements were made, with a minimum interval of 3 min between measurements. The first measurement was discarded and the average of the other two was used.^9^

Two urine collection containers previously identified with the name and the numbers one and two (for the first and second urine collection of the day) were delivered to individuals. They were instructed on collection procedures and storage, and the need to fast for blood collection and clinical exams.

The next day (second stage) participants delivered the urine samples and underwent blood sample collection in a predetermined location. The collected blood and urine samples were identified. The first urine sample was used for sediment analysis and the second was used for creatinine and sodium excretion analysis. The blood was centrifuged and placed in styrofoam containers with ice and transported to the reference laboratory where the analyses were performed. On the same day, weight (in kilograms) was measured on a portable digital scale (Plena(r), Brazil) and height (in meters) with a stadiometer (Alturexata(r), Brazil), both following the recommended techniques.

For anthropometric measurements, the body mass index (BMI) was calculated as the ratio between body weight and the square of the height. The classification recommended by the WHO was used as follows: underweight, BMI < 18.5 kg/m^2^; normal weight, BMI ≥ 18.5 kg/m^2^ and < 25 kg/m^2^; overweight, BMI ≥ 25 kg/m^2^ and < 30 kg/m^2^; and obese, BMI ≥ 30 kg/m^2^.^10^ The waist circumference (WC) was measured at the midpoint between the last rib and the iliac crest at full expiration, using a non-extensible anthropometric tape (Sanny(r), Brazil). The adopted cutoff point was as follows: high risk, WC ≥ 94 cm for men and ≥ 80 cm for women; and very high risk, WC ≥ 102 cm for men and ≥ 88 cm for women.^11^

Measurements of serum creatinine (Jaffé method), fasting plasma glucose (UV Hexokinase, Automated), triglycerides (Enzyme/Trinder, Automated), total cholesterol (Enzyme/Trinder, Automated), high density lipoprotein-cholesterol (HDL-c) (Dextran/Magnesium Sulfate/Sulfate, Automated), low density lipoprotein-cholesterol (LDL-c) (Dextran/Magnesium Sulfate/Sulfate, Automated) were carried out.

The Kawasaki formula,^5^ validated in a Brazilian population,^12^ was used to estimate the sodium excretion. After estimating the excretion of 24-h urinary creatinine (CrPr-24h) and the sodium/creatinine ratio in random urine (Na/CrUr), the total sodium content in 24-h urine (Na-24h) was estimated. The formula is:

23 x 16.3 x {[(Na (mEq/L) in spot urine /Cr (mg/dL) in spot urine x 10] x PrUCr24h}0.5. The predicted values of CrPr-24h (mg) = [(15.12 x weight, kg) + (7.39 x height, cm) - (12.63 x age, years)] - 79.9 (for men); and CrPr-24h (mg) = [(8.58 x weight, kg) + (5.09 x height, cm) - (4.72 x age, years)] - 74.95 (for women).

Renal function was evaluated using serum creatinine and eGFR (mL/min/1.73 m^2^) by using the equation proposed by the Chronic Kidney Disease Epidemiology (CKD-EPI) study: 141 × min(serum creatinine/κ, 1)α × max(serum creatinine/κ, 1)^-1.209^ × 0.993^Age^ × 1.018 if women, × 1.159 if black; with: κ = 0.7 for women and 0.9 for men; α = 0.329 for women and -0.411 for men; min is the minimum serum creatinine or 1; and max indicates the maximum serum creatinine.^13^

The collected data were analyzed using STATA software (STATA Version 14.0, Stata Corporation, College Station, Texas). The descriptive analysis was initially performed, both in the total sample and according to sex. Categorical variables were presented as frequencies and percentages and numerical as means and standard deviations (mean ± SD). The normality of the numerical variables was assessed using the Shapiro-Wilk test.

To compare differences in the means by sex, an unpaired *t*-test or Mann-Whitney test was used. The level of significance was 5%. A multivariate linear regression model was adjusted to determine the relation between GFR and sodium excretion. In the regression analysis, the numeric independent variables have been rescaled by subtracting their mean and dividing by their standard deviations. This standardization was carried out to facilitate comparability of the relative importance of independent variables in the model.

The local ethics committee approved the PREVRENAL study, which was carried out according to their rules.

## RESULTS

A total of 1,211 individuals of African descent were included in the final sample. The characteristics of the total sample grouped by sex (men and women) is shown in Table 1. The mean age was 37.5 ± 11.7 years; 52.8% were women, 89.3% declared themselves as black or brown, and 58.6% had less than 8 years of schooling. The prevalence of alcoholic beverage and cigarette consumption was 46.4 and 10.2%, respectively. Regarding anthropometric evaluation, 13.3% of respondents were obese based on BMI, and 30.6% had a high risk of cardiovascular disease according to WC.

**Table 1:** Demographic, socioeconomic, and anthropometric characteristics of African descendants of Alcântara - MA, 2013

Variables	Total n (%)	Men n (%)	Women n (%)	p-value
Skin color				0.789
White	124 (10.2)	55 (44.3)	69 (55.7)	
Black or brown	1081 (89.3)	514 (47.6)	567 (52.4)	
Other	6 (0.5)	3 (50.0)	3 (50.0)	
Highest education				0.157
≤ 8 years	709 (58.6)	347 (48.9)	362 (51.1)	
> 8 years	502 (41.4)	225 (44.8)	277 (55.2)	
Income (× minimum wage)				0.012
No fixed income	606 (50.1)	303 (50.0)	303 (50.0)	
Up to 1	429 (35.4)	185 (43.1)	244 (56.9)	
> 1 and ≤ 2	136 (11.2)	62 (45.6)	75 (54.4)	
> 2	40 (3.3)	22 (55.0)	18 (45.0)	
Smoking				< 0.001
Yes	123 (10.2)	96 (78.1)	27 (21.9)	
Non-smoker or former smoker	1088 (89.8)	476 (43.8)	612 (56.2)	
Alcohol consumption				< 0.001
Yes	562 (46.4)	359 (63.9)	203 (36.1)	
Non-drinker or former drinker	649 (53.6)	213 (32.9)	436 (67.1)	
BMI (kg/m^2^)				< 0.001
Underweight	25 (2.1)	12 (63.9)	13 (52.0)	
Normal weight	620 (51.2)	365 (58.9)	255 (41.3)	
Overweight	404 (33.4)	165 (40.8)	239 (59.2)	
Obese	161 (13.3)	29 (18.0)	132 (82.0)	
WC (cm)				< 0.001
Normal	649 (53.6)	491 (75.6)	158 (24.4)	
High risk	191 (15.8)	531 (27.8)	138 (72.2)	
Very high risk	371 (30.6)	28 (7.6)	343 (92.4)	

BMI: body mass index; WC: waist circumference.

Women were significantly more likely than men to be overweight or obese according to BMI and had more WC values within the risk group. However, higher income, smoking, and alcohol use were more frequent among men (Table 1).

The prevalence of reduced (<60) and elevated (>120 mL/min/1.73m^2^) GFR were 0.5% and 32.9%, respectively (data not shown in table). The average urinary sodium excretion of the total sample was 204.6 ± 86.2 mmol/day. Male subjects had, on average, higher SBP (128.6 ± 16.5 mm Hg vs 122.6 ± 20.0 mm Hg; p < 0.001) and serum creatinine (0.8 ± 0.1 mg/dL vs 0.6 ± 0.1 mg/dL; *p* < 0.001) values than female subjects did. On the contrary, women had higher values for total cholesterol, HDL-c, LDL-c, and estimated GFR by CKD-EPI equation than male subjects did (Table 2).

**Table 2:** Characteristics according to clinical and biochemical variables of African descendants of Alcântara - MA, 2013

Variables	Total	Men	Women	p-value
SBP (mmHg)	125.4 ± 18.7	128.6 ± 16.5	122.6 ± 20.0	< 0.001
DBP (mmHg)	76.0 ± 11.3	75.5 ± 11.5	76.5 ± 11.2	0.205
Fasting glucose (mg/dL)	100.4 ± 26.5	99.5 ± 22.6	102.2 ± 29.6	0.369
Total cholesterol (mg/dL)	186.6 ± 43.8	176.8 ± 38.6	195.3 ± 46.4	< 0.001
HDL-c (mg/dL)	49.0 ± 17.9	47.3 ± 13.9	50.5 ± 20.6	< 0.001
LDL-c (mg/dL)	114.0 ± 36.3	106.5 ± 32.8	120.5 ± 37.9	< 0.001
Triglycerides (mg/dL)	120.6 ± 79.0	117.7 ± 76.9	123.1 ± 80.8	0.149
Serum creatinine (mg/dL)	0.7 ± 0.2	0.8 ± 0.1	0.6 ± 0.1	< 0.001
GFR (mL/min/1.73m^2^)	111.8 ± 15.3	110.6 ± 14.6	112.8 ± 15.9	< 0.001
Urinary sodium excretion (mmol/day)	204.6 ± 86.2	206.5 ± 77.5	202.8 ± 93.9	0.144

Values are mean ±SD; SBP: systolic blood pressure; DBP: diastolic blood pressure; HDL-c: high density lipoprotein cholesterol; LDL-c: low density lipoprotein-

According to multivariate linear regression, GFR was independently correlated with sodium excretion (β = 0.11; *p* < 0.001), age (β = -0.67; *p* < 0.001), female sex (β = -0.20; *p* < 0.001), and BMI (β = -0.09; *p* < 0.001) (Table 3).

**Table 3:** Multivariate linear regression analysis coefficients for glomerular filtration rate estimated by CKD-EPI Equation

Variables	Univariate			Multivariate		
	Β	p-value	CI 95%	Β	p-value	CI 95%
Age	0.49	< 0.001	0.48 - 0.52	-0.67	< 0.001	-0.72 - -0.62
Female sex	0.86	0.011	0.77 - 0.97	-0.20	< 0.001	-0.29 - -0.12
BMI	0.81	< 0.001	0.77 - 0.86	-0.09	< 0.001	-0.14 - -0.04
Fasting glucose	0.84	< 0.001	0.80 - 0.90			
Average SBP	0.74	< 0.001	0.70 - 0.78			
Average DBP	0.74	< 0.001	0.70 - 0.78			
Urine Na excretion	1.08	0.008	1.02 - 1.15	0.11	< 0.001	0.07 - 0.15

β = model coefficient; CI: confidence interval; BMI: body mass index; SBP: systolic blood pressure; DBP: diastolic blood pressure.

## DISCUSSION

In the present study, an average of 204.6 ± 86.2 mmol/day of sodium excretion was observed among Brazilians of African ancestry, representing a salt consumption of 11.9 g/day (4.7 g of sodium/day). This value is similar to that reported for the overall Brazilian population, which is approximately 11.4 g/day of salt (4.5 g of sodium/day)^14^ and worldwide, ranging from 9 to 12 g per person per day.^15^ This value exceeds by more than two times the WHO recommendation, which is 5 g of salt/day (2.0 g of sodium/day).^16^

Other studies reported lower values. The Prevention of Renal and Vascular End-stage Disease (PREVEND) study in the Netherlands with 7,543 adults yielded an average 24-h sodium excretion of 142 ± 51 mmol/day, which corresponds to a daily intake of 8.2 g of salt/day (3.3 g of sodium/day).^17^ Asian individuals presented even lower values, albeit still exceeding WHO recommendations16 with a mean value of 125 ± 53.4 mmol/day (7.3 g of salt/day),^18^ highlighting the need for additional efforts to reduce salt consumption.

In the present study, we observed a positive association between sodium excretion in a single urine sample and GFR. It should be noted that the average GFR values in the population were within normal parameters; only 6 patients (0.5%) showed a reduced GFR (< 60 mL/min/1.73 m^2^), while 32.9% showed a GFR > 120 mL/min/1.73m^2^. Thus, elevated sodium excretion occurred in subjects with relatively preserved renal function. The positive correlation between GFR and very high levels of sodium excretion could lead to hyperfiltration scenarios and, consequently, to harmful effects.

A study conducted by Park et al.,^19^ found an association between glomerular hyperfiltration and increased mortality from all causes in an apparently healthy population. The study also pointed to the possibility of using glomerular hyperfiltration as a new marker for mortality.

The literature suggests that African-Americans are more prone to retention of sodium and to salt-sensitive hypertension than Caucasians.^20^ According to Price et al., even healthy African-Americans show a 10% lower in renal plasma flow compared to age-matched Caucasians, possibly due to the action of an activated intrarenal renin-angiotensin system (RAS).^21^ Brenner et al. also point out that people of African descent have fewer nephrons compared to Caucasians.^22^

Moreover, high sodium intake has been linked to CKD progression. Deficient sodium excretion is often associated with compromised renal function, causing high blood pressure and proteinuria, glomerular hyperfiltration, and reduced response to intrarenal RAS blockade.^2^^,^ 23

Data from a prospective cohort study conducted on stage 3 kidney failure patients seen in primary care showed that CKD patients who reduced their sodium intake showed a decrease in BP, proteinuria, and pulse-wave speed over a 1-year period. A subgroup analysis that included only albuminuric participants showed an improvement in albuminuria only in those who decreased their sodium intake.^24^ Therefore, it has been recommended to reduce sodium intake in adults to < 2 g per day (corresponding to < 5 g of sodium chloride) to preserve renal function.^25^

The present study also found a negative association between age and GFR. It is known that age should be considered when establishing GFR reference values, as renal function decreases with age.^26^ Soares et al.^27^ showed that GFR begins to decrease significantly after 45 years of age, with a significant inverse correlation between age and GFR (r = -0.33, *p* < 0.001).

Granerus and Aurell^28^ analyzed data from eight studies conducted between 1950 and 1980, finding a decline in GFR of up to 4 mL/min per decade for ages <50 years, and up to 10 mL/min for ages >50 years. Subsequently, Poggio et al.^29^ showed that GFR decreases at a rate of approximately 4 mL/min per decade in younger (<45 y) individuals.

In the present study, GFR was higher in women, corroborating a study by Carrero^30^ that highlighted the influence of sex in CKD progression. The possible explanations for sex-influenced GFR decreases include cultural, social, and environmental differences such as adherence to treatment or disease perception, and biological differences, such as genetic and hormonal factors.^31^

Obesity also had an impact on GFR. Our findings showed that BMI correlated negatively with GFR, unlike other previously published studies. Glomerular hyperfiltration has been suggested as a possible mechanism linking obesity to CKD. Ogna et al.^32^ evaluated 1339 participants and identified a prevalence of overweight and obesity of 32.2 and 14.2%, respectively. The mean CrCl in overweight individuals was 110 [87-136] mL/min and 124 [97-150] mL/min in obese subjects (*p* < 0.001). The prevalence of glomerular hyperfiltration increased with increasing categories of BMI (10.4, 20.8 and 34.7%, respectively, p <0.001). This positive association remained significant after adjustment for other known CKD risk factors, suggesting that glomerular hyperfiltration may represent an early renal phenotype in obesity.

Another study conducted in Melbourne with individuals aged between 18 and 57 years showed that 85.6% were classified as overweight or obese according to BMI and a positive association between eGFR and BMI (r = 0.23, *p* = 0.02) and waist circumference (r = 0.23, *p* = 0.02) was observed, although none of the anthropometric measures were independently related to eGFR. There was a tendency for BMI to interact with eGFR, particularly in the overweight group, although it was not statistically significant.^33^ In agreement with our findings, a cross-sectional study of 1,100 Chinese subjects showed a significantly decreased eGFR that was negatively correlated to BMI regardless of the presence of diabetes or high blood pressure. Every 1.0 kg/m2 increase in BMI led to a decrease of 0.5 mL/min/1.73m2 in eGFR.^34^ Another study conducted in a representative sample of the British population demonstrated that the risk of CKD was 2.5 times higher in obese participants compared to normal-weight participants in the adjusted model (BMI = 30.0-39.9 kg/m2: Adjusted OR = 2.78 (95% CI=1.75-4.43); BMI ≥ 40.0 kg/m2: Adjusted OR = 2.68 (95% CI=1.05-6.85).^35^

A cohort study performed with 3,376,187 American individuals with eGFR > 60 mL/min/1.73m^2^ also evaluated its association with BMI over different age groups. It was shown that 8.1% of patients showed a rapid decline in renal function (> 5 mL/min/1.73m^2^), exhibiting a consistent U-shaped association with BMI, which was more prominent with increasing age; patients younger than 40 years were an exception, as BMI did not appear to be a predictor of compromised renal function.^36^

The estimate of sodium intake from a random urine sample, at a population level, is increasingly used as a convenient and affordable alternative, although 24-h urine collection is the "gold standard". Nevertheless, random urine samples may indicate poorly 24-h urinary excretion due to individual variations3, which is a limitation.

Many formulas have been proposed to convert sodium in random urine sample into a 24-h excretion estimation by using creatinine to adjust for urinary concentration. A study in different populations showed a larger interclass correlation coefficient between the estimated and measured excretion of sodium using Kawasaki's equation compared to INTERSALT and Tanaka's Equations.^37^ In Brazil, Mill et al. developed a study to validate the Tanaka and Kawasaki equations.^12^

The strengths of this study include the large representative sample and the random selection of participants, who belonged to a vulnerable population. This was the first population-based study on African-descent communities of Maranhão and Brazil that evaluated eGFR and sodium excretion.

A limitation to the study is the cross-sectional nature of urine sampling. We did not use the gold standard for measuring sodium excretion, but rather an estimating equation validated in Brazil.

Although there is individual variation in sodium excretion during the day, a random sample tends to underestimate or overestimate its excretion. However, the estimate of urinary sodium can be a means to monitor dietary sodium intake in population studies, and in low-income individuals, where 24-h collection can be logistically difficult for several reasons.^4^

## CONCLUSIONS

The present study showed a low occurrence of reduced GFR and high sodium excretion in this ethnic group. Sodium excretion was the only variable that correlated positively with GFR, indicating that high levels can contribute to hyperfiltration scenarios and their potential future negative consequences. It is suggested that the measurement of sodium excretion should be incorporated as a CKD- and cardiovascular-disease-preventive measure in populations with similar clinical characteristics.
